# Reduced Levels of Proteasome Products in a Mouse Striatal Cell Model of Huntington’s Disease

**DOI:** 10.1371/journal.pone.0145333

**Published:** 2015-12-21

**Authors:** Sayani Dasgupta, Michael A. Fishman, Hana Mahallati, Leandro M. Castro, Alexandre K. Tashima, Emer S. Ferro, Lloyd D. Fricker

**Affiliations:** 1 Department of Molecular Pharmacology, Albert Einstein College of Medicine, 1300 Morris Park Ave, Bronx, New York, 10461, United States of America; 2 São Paulo State University (UNESP), Experimental Campus on the São Paulo Coast, São Vicente, 11330–900, SP, Brazil; 3 Department of Biochemistry, Escola Paulista de Medicina, Federal University of Sao Paulo, Sao Paulo, SP, 04023–901, SP, Brazil; 4 Department of Pharmacology, Biomedical Science Institute, University of São Paulo, São Paulo, 05508–000, SP, Brazil; 5 Department of Neuroscience, Albert Einstein College of Medicine, 1300 Morris Park Ave, Bronx, New York, 10461, United States of America; National Center of Neurology and Psychiatry, JAPAN

## Abstract

Huntington’s disease is the result of a long polyglutamine tract in the gene encoding huntingtin protein, which in turn causes a large number of cellular changes and ultimately results in neurodegeneration of striatal neurons. Although many theories have been proposed, the precise mechanism by which the polyglutamine expansion causes cellular changes is not certain. Some evidence supports the hypothesis that the long polyglutamine tract inhibits the proteasome, a multiprotein complex involved in protein degradation. However, other studies report normal proteasome function in cells expressing long polyglutamine tracts. The controversy may be due to the methods used to examine proteasome activity in each of the previous studies. In the present study, we measured proteasome function by examining levels of endogenous peptides that are products of proteasome cleavage. Peptide levels were compared among mouse striatal cell lines expressing either 7 glutamines (ST*Hdh* ^Q7/Q7^) or 111 glutamines in the huntingtin protein, either heterozygous (ST*Hdh* ^Q7/Q111^) or homozygous (ST*Hdh* ^Q111/Q111^). Both of the cell lines expressing huntingtin with 111 glutamines showed a large reduction in nearly all of the peptides detected in the cells, relative to levels of these peptides in cells homozygous for 7 glutamines. Treatment of ST*Hdh* ^Q7/Q7^ cells with proteasome inhibitors epoxomicin or bortezomib also caused a large reduction in most of these peptides, suggesting that they are products of proteasome-mediated cleavage of cellular proteins. Taken together, these results support the hypothesis that proteasome function is impaired by the expression of huntingtin protein containing long polyglutamine tracts.

## Introduction

Huntington’s disease (HD) is an autosomal dominant neurodegenerative disorder caused by expansion of CAG trinucleotide repeats within exon 1 of the *HTT* gene that encodes huntingtin protein [1–3]. Typically, the number of residues in the polyglutamine (polyQ) tract in huntingtin protein averages between 16–20 in the normal population and >35 in people with HD. Symptoms of HD include motor impairments (chorea, incoordination, bradykinesia), cognitive decline, and emotional disorders. Often, death occurs within 15–20 years from onset of symptoms. Although huntingtin protein is expressed throughout the brain, neuronal cell death is most prominent in the striatum, with less severe pathology seen in the cortex and thalamus [1–4].

Many theories have been proposed to explain the neurotoxicity of long polyQ tracts within the huntingtin protein and other related polyQ diseases, such as problems due to aggregation of the protein with long polyQ sequences [2]. However, it has been argued that the aggregates are protective, serving to sequester the long polyQ proteins and prevent toxicity [5,6]. A potential mechanism for toxicity of long polyQ proteins is through inhibition of the proteasome, a multicatalytic protein complex that plays an essential role in intracellular protein degradation. The conventional dogma is that the proteasome cleaves proteins into peptides of 2–24 amino acids, acting primarily on regions with hydrophobic or charged amino acids, and with lower activity at Q residues [7,8]. Long stretches of polyQ were proposed to inhibit the proteasome and thereby lead to cellular toxicity [9]. Some studies found evidence in support of this theory, while other studies did not [9–29]. These previous studies used a variety of experimental approaches to assay proteasome activity; some measured cleavage of fluorogenic peptides that are proteasome substrates while others measured levels of reporter proteins expressed with or without a degradation signal. Each of these approaches have yielded insights about the role of proteasome function in HD, but each method explores a different component of the ubiquitin-proteasome pathway and is limited by the ability to examine the degradation of only a few select substrates. A more accurate understanding of endogenous proteasome function can come from measuring levels of endogenous products of the proteasome, i.e. intracellular peptides.

Mass spectrometry based peptidomic studies have detected a large number of protein-derived peptides from cell lines and animal tissues [30–32]. The vast majority of these intracellular peptides are proteasome products, based on studies performed with proteasome inhibitors in cell lines [33–35]. In the present study, we have used a quantitative peptidomics method to detect and measure the levels of proteasome products in HD model cell lines, ST*Hdh* ^Q7/Q7^, ST*Hdh* ^Q7/Q111^ and ST*Hdh* ^Q111/Q111^ (which we refer to as Q7Q7, Q7Q111 and Q111Q111 respectively). These cells are striatal progenitor cells derived from a knock-in mouse model of HD and endogenously express full-length huntingtin with either short (7) or long (111) polyQ repeats [36]. We find that the levels of a large number of intracellular peptides are decreased in the cell lines expressing huntingtin with Q111, relative to the Q7 cells. Most of the peptides found to be decreased in the Q111 cells are proteasome products, based on the finding that treatment of Q7 cells with proteasome inhibitors (epoxomicin, bortezomib) causes a similar decrease in peptide levels. Taken together, these results support the hypothesis that proteasome function is impaired by expression of huntingtin with long polyQ repeats.

## Materials and Methods

### Reagents

Q7Q7, Q7Q111 and Q111Q111 cells were obtained from Dr. Erik Snapp (Albert Einstein College of Medicine) and cultured as described [36]. High glucose Dulbecco’s Modified Eagle’s Medium (DMEM), Dulbecco’s Phosphate Buffered Saline (DPBS), penicillin-streptomycin, and M-MLV Reverse Transcriptase were purchased from Invitrogen. Fluorescent substrates containing the 7-amino-4-methylcoumarin (AMC) group were procured from Bachem. Hydroxylamine, G-418, glycine, sodium hydroxide, dibasic sodium phosphate, dimethyl sulfoxide (DMSO), and bestatin were purchased from Sigma. Hydrochloric acid, mass spectroscopy grade trifluoroacetic acid (TFA), and C-18 spin columns were purchased from Pierce Thermo Scientific. Other reagents and their sources were puromycin (Tocris Bioscience), TriPure Reagent (Roche), Power SYBR Green Master Mix (Applied Biosystems), acetonitrile (Fisher), fetal bovine serum (Seradigm), epoxomicin (Calbiochem), and bortezomib (LC Laboratories). The isotopic labeling reagents 4-trimethylammoniumbutyryl-N-hydroxysuccinimide (TMAB-NHS) containing either 0, 3, 6, or 9 atoms of deuterium (D0, D3-, D6-,and D9-, respectively) or 9 atoms of deuterium and three ^13^C atoms (D12-) were synthesized as described [37,38].

### Proteasome activity assay

Q7Q7, Q7Q111 and Q111Q111 cells were lysed by sonication in 50 mM Tris-HCl buffer, pH 7.5, containing 40 mM KCl, 5 mM MgCl_2_, 0.5 mM ATP, and 1 mM DTT (Buffer 1). The cell extract was incubated with Buffer 1 and 100 μM final concentration of the proteasome substrate succinyl-Leu-Leu-Val-Tyr-7-amino-4-methylcoumarin (Suc-Leu-Leu-Val-Tyr-AMC) or acetyl-Arg-Leu-Arg-7-amino-4-methylcoumarin (Ac-Arg-Leu-Arg-AMC). After incubation at 37°C for 1 hour, proteasome activity was quantified by fluorescence measurement of the AMC product (380 nm excitation, 460 nm emission). Protein concentration was measured by the bicinchoninic acid protein assay kit (Pierce Thermo Scientific) as described in the protocol provided by the manufacturer.

### Aminopeptidase activity assay

Q7Q7, Q7Q111, and Q111Q111 cells were lysed by sonication in Buffer 1, combined with Leu-AMC (final concentration 100 μM), and incubated at 37°C for 1 hour. Enzyme activity was quantified by fluorescence measurement of the AMC product (380 nm excitation, 460 nm emission). Protein concentration was measured by the bicinchoninic acid protein assay kit. For studies examining the sensitivity of the enzyme to peptidase inhibitors, the cell lysate was preincubated with bestatin or puromycin for 30 minutes prior to the addition of substrate.

### Cell culture and peptide extraction

Q7Q7, Q7Q111, and Q111Q111 cells were grown to 80–90% confluence in 15 cm cell culture plates with DMEM supplemented with 10% fetal bovine serum, penicillin-streptomycin and 0.4 mg/mL G-418. A single plate was used for each cell line. At the start of the experiment, media were removed from the plates and cells were washed four times with DPBS to remove traces of serum. After centrifugation at 800 X *g* for 5 min, the cell pellet was resuspended in 1 mL of 80°C water and incubated in a water bath at 80°C for 20 min. The mixture was again centrifuged (13,000 X *g*, 30 min, 4°C) and stored at -80°C overnight. For peptide extraction, the samples were centrifuged again. The lysate was cooled on ice and acidified with HCl to a final concentration of 10 mM. After 15 min incubation on ice, the lysate was centrifuged at 13,000 X *g* for 30 min at 4°C. Sodium phosphate (250 μL of 0.4 M, pH 9.5) was added to the supernatant and the mixture was stored at -80°C until labeling.

### Proteasome inhibitor treatment

Q7Q7 cells were grown to 80–90% confluence in 15 cm cell culture plates as described above. A single plate of cells was used for each group. At the beginning, media were removed and cells washed with DPBS. This was followed by addition of serum-free media containing the proteasome inhibitors (dissolved in DMSO, for a final concentration of 0.05%) or 0.05% DMSO alone. Each experiment consisted of two DMSO controls and two treated groups of cells. The cells were incubated at 37°C for 45 min, following which media containing the inhibitor were removed, cells were washed twice with DPBS and centrifuged at 800 X *g* for 5 min. The wash buffer was supplemented with the appropriate inhibitor at the same concentration used for the treatment. The length of the wash procedures was 15 min, and the total time of exposure of cells to epoxomicin or bortezomib was therefore 60 min. Cell pellets were resuspended in 80°C water, incubated at 80°C for 20 min, and peptides extracted as described above.

### Quantitative peptidomics

Quantitative peptidomics was performed using the differential isotopic labeling strategy and trimethylammonium butyrate (TMAB) activated with N-hydroxysuccinimide (NHS), as described earlier [37]. Each group within an experiment was labeled with a different isotopic tag (D0-, D3-, D6-, D9- or D12-TMAB-NHS). The labels were dissolved in DMSO to a concentration of 0.4 mg/μL and 7.5 mg of label was used per plate of cells. At the start of the experiment, the pH of the peptide extract was adjusted to 9.5 with 1 M NaOH. Labeling was performed over 8 rounds; 2.3 μL of the label was added to the extract every 20 min. The pH was measured between each round and if necessary, brought back to 9.5. After the final round of labeling, the pH was adjusted to 9.5 again and the extracts were incubated at room temperature for 90 min. To quench any unreacted label, 30 μL of 2.5 M glycine was added and the mixture incubated 40 min at room temperature. The labeled extracts from each experiment were pooled as shown in S1 Fig and filtered through Amicon Ultracel-10K units. Thereafter, 30 μL of 2 M hydroxylamine was added over three rounds to hydrolyze TMAB-labeled tyrosine. This was done to ensure that only N-terminal amines and lysine side-chain amines of peptides were TMAB-labeled. The pH was adjusted to 9.0 before each addition of hydroxylamine. The samples were desalted through C-18 spin columns. Peptides were eluted using 160 μL of 0.5% TFA and 70% acetonitrile, freeze-dried in a vacuum centrifuge and stored at -80°C until analysis by mass spectrometry.

Liquid chromatography-mass spectrometry (LC-MS) analysis was performed on a Synapt G2 mass spectrometer coupled to a nanoAcquity capillary LC system (Waters, Milford, MA, USA). The peptide mixture was desalted online for 5 min at a flow rate of 8 μL/min of phase A (0.1% formic acid) using a Symmetry C18 trapping column (5 μm particles, 180 μm inner diameters, 20 mm length; Waters). The mixture of trapped peptides was subsequently separated through a BEH 130 C18 column (1.7 μm particles, 100 μm inner diameter, 100 mm length; Waters) and peptides eluted with a gradient of 7–65% of phase B (0.1% formic acid in acetonitrile) over 42 minutes at a flow rate of 275 nL/min. The column eluate was directly sampled by electrospray ionization and spectra were acquired for 0.2 seconds over the m/z range 300–1600. The three most intense ions exceeding base peak intensity threshold of 2500 counts were selected and dissociated by 15- to 60-eV collisions with argon for 0.2 seconds. The typical conditions consisted of a capillary voltage of 3.0 kV, a block temperature of 70°C, and a cone voltage of 50 V. The dynamic peak exclusion window was set to 90 seconds.

### Data analysis

The MS spectra were analyzed using MassLynx, version 4.0 (Waters). In the first stage of the analysis, the MS spectra were manually examined for peak sets reflecting peptides with the various isotopic forms of the TMAB tags. The m/z values of each monoisotopic peak set were logged into a spreadsheet along with the peak intensity of the most intense peak in each set (usually either the mono-isotopic peak or the peak containing one ^13^C atom). In the second stage, peptides were identified by MS/MS sequencing by the Mascot program followed by manual verification as described [37,39,40]. The database searched was SwissProt_AC AC_20150324, limited to mouse sequences (547964 sequences; 195174196 residues). No cleavage site was specified. Modifications included the TMAB labels (termed ‘GIST’ in Mascot) and also N-terminal protein acetylation, methionine oxidation, and cyanylation of Cys. All Mascot results were manually checked to exclude false positives, based on a number of criteria that have been previously established [39,40]. Those peptides detected in the first stage of the analysis of MS spectra but not identified in the second stage, either because there was no MS/MS data or because the data was inconclusive, are included in S1 Table along with the identified peptides.

### RNA Isolation and Analysis

mRNA transcript levels were analyzed in Q7Q7 and Q111Q111 cells by reverse transcription followed by quantitative PCR (RT-qPCR). Twenty four hours after plating, media were replaced with media containing 0.05% DMSO. One hour later, media were removed again, cells were washed with DPBS and the plates received fresh media. After 3 hours, RNA was extracted with TriPure Reagent according to the manufacturer’s protocol. cDNA was synthesized with M-MLV Reverse Transcriptase according to the manufacturer’s protocol. cDNA, forward and reverse primers, and Power SYBR Green Master Mix were incubated at 95°C for 10 min and then amplified for 45 cycles (95°C for 15 s, 60°C for 1 min) using a LightCycler 480 (Roche). Expression levels were normalized to those of *Actb*. See S2 Fig for primer sequences.

## Results

**Fig 1:**
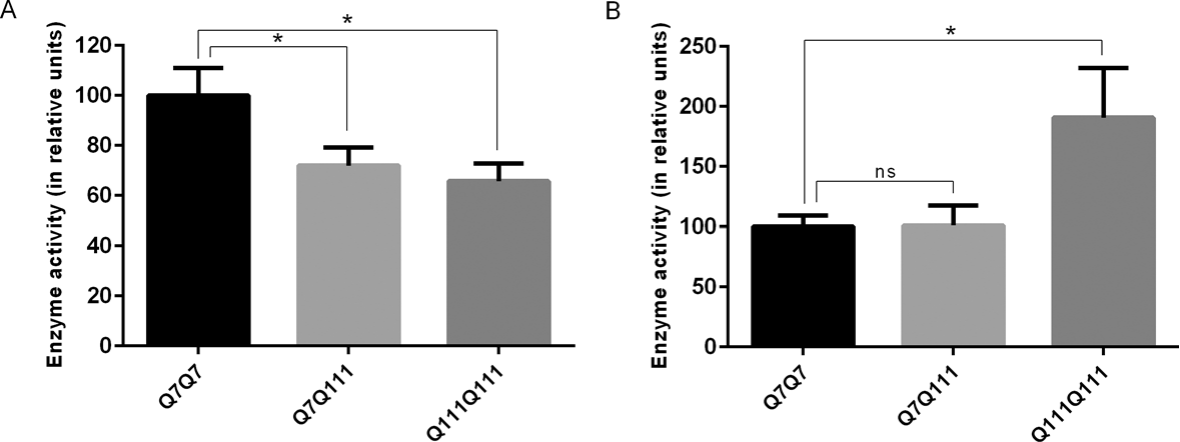
Measurement of proteasome activity in Q7Q7, Q7Q111 and Q111Q111 cells. Cell extracts were incubated with fluorogenic peptide substrates for 1 hour at 37°C, following which enzyme activity was determined by fluorescence measurement of AMC. The activity in Q7Q111 and Q111Q111 cells is expressed as percent enzyme activity relative to the Q7Q7 cells. Activities of the chymotrypsin-like (β5) and trypsin-like (β1) proteolytic subunits were measured by cleavage of Suc-Leu-Leu-Val-Tyr-AMC (A) and Ac-Arg-Leu-Arg-AMC (B) respectively. The error bars show standard error of mean (n = 10 for Suc-Leu-Leu-Val-Tyr-AMC and n = 5 for Ac-Arg-Leu-Arg-AMC). Statistical analysis was performed using Student’s t-test: *, p ≤ 0.05; ns, no significant difference (p > 0.05) versus Q7Q7 cells.

Q7Q7, Q7Q111, and Q111Q111 cell lines were previously established from wild-type and Hdh(Q111) knock-in embryos, as described by Trettel et al [36]. A previous study compared Q7Q7 and Q111Q111 cell extracts using assays for three different proteasome subunits and found a decrease in chymotrypsin-like activity and caspase-like activity, but an increase in trypsin-like activity in the Q111Q111 cells [20]. To confirm this result, and to extend the study to heterozygous Q7Q111 cells, we assayed lysates from the three cell lines for their ability to cleave fluorogenic peptide substrates Suc-Leu-Leu-Val-Tyr-AMC and Ac-Arg-Leu-Arg-AMC that measure chymotrypsin-like and trypsin-like proteolytic activities of the proteasome, respectively. Both cell lines expressing Q111 showed a significant ~30% decrease in chymotrypsin-like activity compared to the Q7Q7 cells (Fig 1A). The trypsin-like activity of Q111Q111 cells was nearly double that of the Q7Q7 cells, though Q7Q111 cells did not show a significant difference (Fig 1B). Our results with the Q7Q7 and Q111Q111 cells are comparable to the previous report using a similar assay on these cell lines [20], and suggest a complex effect where chymotrypsin-like activity is impaired while trypsin-like activity is activated.

**Fig 2:**
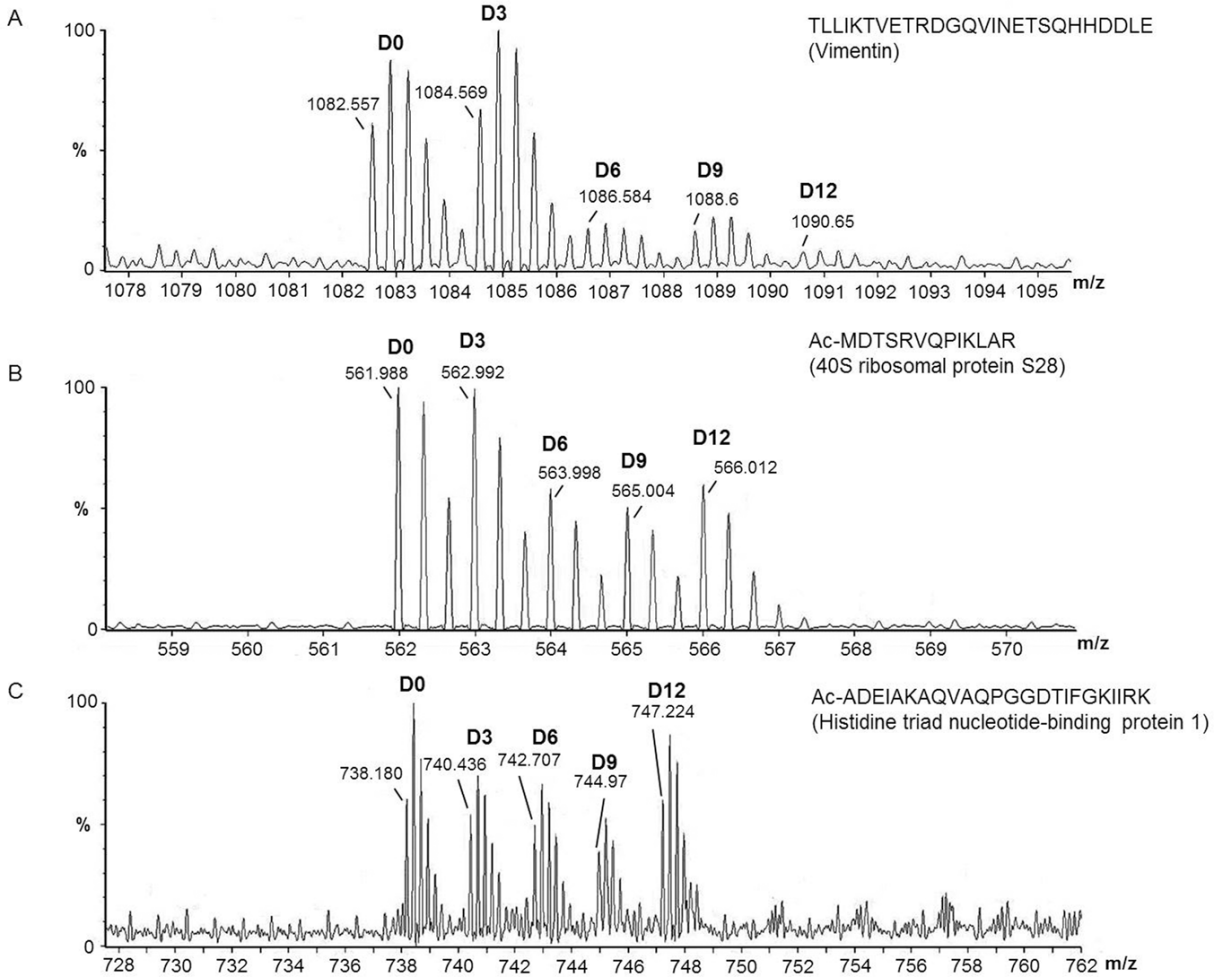
Representative MS data from peptidomics experiment comparing peptide levels between Q7Q7, Q7Q111 and Q111Q111 cells. In this experiment, the two replicates of Q7Q7 cells were labeled with D0- and D3-TMAB-NHS, one replicate of the Q7Q111 cells was labeled with D9-TMAB-NHS and two replicates of Q111Q111 cells were labeled with D6- and D12-TMAB-NHS. A: Example of a peptide that is present in the Q111-expressing cell lines at levels much lower than in Q7Q7 cells. This peptide was identified by MS/MS analysis as TLLIKTVETRDGQVINETSQHHDDLE, derived from vimentin. B: Example of a peptide that is present in the Q111-expressing cell lines at levels slightly lower than in Q7Q7 cells. This peptide was identified by MS/MS analysis as Ac-MDTSRVQPIKLAR, derived from the 40S ribosomal protein S28. C: Example of a peptide that is present in all cell lines at comparable levels. This peptide was identified by MS/MS analysis as Ac-ADEIAKAQVAQPGGDTIFGKIIRK, derived from histidine triad nucleotide-binding protein 1.

To determine if proteasome activity towards endogenous substrates was affected, we used a quantitative peptidomics technique to examine relative levels of intracellular peptides in the three cell lines. In this technique, cells are heat inactivated to block post-extraction degradation, the peptides are extracted, labeled with stable isotopic tags, and analyzed by LC-MS [37,39–42]. In each experiment, we used 4–5 distinct isotopic tags so that all three cell lines could be compared in each LC-MS run, and so that replicates of some of the lines could be included in each run. Altogether, 4 LC-MS runs were performed, with a total of 6 replicates of the Q7Q7 cells, 5 replicates of the Q7Q111 cells, and 6 replicates of the Q111Q111 cells (S1 Fig). The MS spectra were analyzed to identify groups of peaks corresponding to isotope-tagged peptides. Relative levels of peptides were quantified by comparing the peak intensity for each of the isotopic peaks in the Q7Q111 and Q111Q111 cells to the peak intensity in the Q7Q7 cells. Fig 2 shows representative MS data comparing peptide levels between Q7Q7, Q7Q111, and Q111Q111 cells. Panels A and B are spectra of peptides that are present at lower levels in the cells expressing Q111 huntingtin relative to levels in the Q7Q7 cells. In these examples, there is no major difference between the peak intensity of the peptide in heterozygous Q7Q111 cells (labeled with D9-TMAB) versus homozygous Q111Q111 cells (labeled with D6- and D12-TMAB), and all three replicates show lower peak intensities than Q7Q7 cells (labeled with D0 and D3-TMAB). Panel C is a spectrum of a peptide that shows no major difference among the three cell lines.

Altogether, 341 distinct peptides were identified in at least one of the LC-MS runs, and over 400 additional peptides were detected in MS but not identified by MS/MS. The 341 identified peptides are derived from 80 distinct genes. S1 Table lists every peptide detected in the MS analysis, with a separate row for every peak group m/z and relative levels for the cell lines compared in each run. Since the peptide levels are expressed as a relative ratio, any peptide found in Q7Q7 cells but not detected in Q7Q111 or Q111Q111 cells was listed as <0.20, reflecting the typical signal to noise ratio of 5:1. In some cases, the signal to noise was much higher than 5:1, but the lower limit of <0.20 was still used. Many of the peptides were identified by MS/MS sequence analysis using manual evaluation and standard criteria [37,39,40]. All peptides (identified and unidentified) are included in S1 Table; all further analysis focused only on the identified peptides.

**Fig 3:**
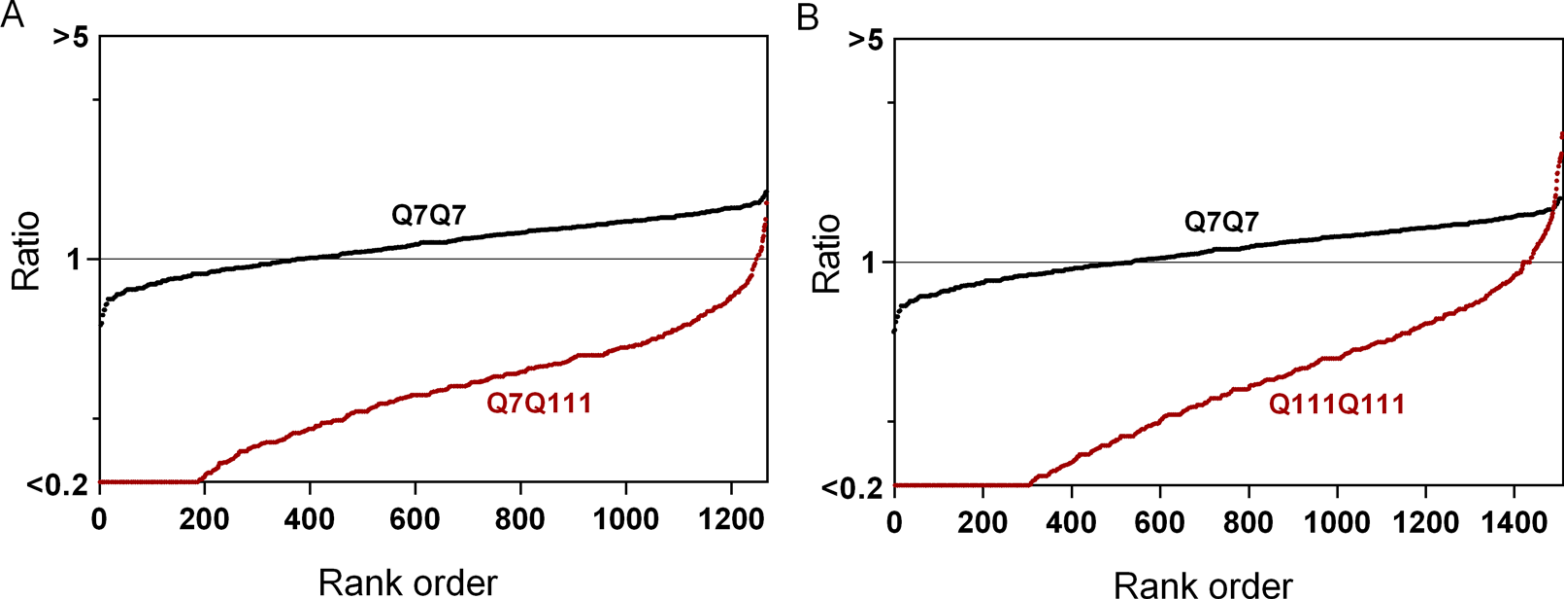
Summary plots of the peptidome of Q7Q7, Q7Q111 and Q111Q111 cells. The relative levels of all peptides identified by MS/MS analysis in the Q7Q111 (A) and Q111Q111 (B) cells were compared to the average level of the peptide in Q7Q7 cells. The *y*-axis represents relative peptide levels (log-scale) and the *x*-axis represents the rank order of peptides sorted according to the relative level. For those peptides detected multiple times (different charge states and/or tag numbers), the relative ratio for each form was considered separately. If the ratio was <0.20, the value was capped at 0.20 to reflect the typical signal to noise ratio. The red circles represent the ratio of each replicate of the identified peptides in Q7Q111 or Q111Q111 cells, expressed relative to the level in Q7Q7 cells for that LC-MS run. The black circles represent the ratio of each Q7Q7 replicate expressed relative to the average level of Q7Q7 replicates in that LC-MS run (i.e. run 1 and 2; the other two runs did not include replicates of Q7Q7 cells).

To visualize the results, summary plots were generated to show the levels of peptides in the Q7Q111 and Q111Q111 cells in comparison to the level in Q7Q7 cells (Fig 3). In these plots the *y*-axis shows the ratio of each biological replicate for every identified peptide. The ratio was sorted from low to high and plotted; the *x*-axis represents the rank order of the peptides. Most of the peptides in the Q7Q111 and Q111Q111 cells show a large decrease relative to their levels in the Q7Q7 cells (Fig 3). The different levels of peptides in the Q111-expressing cell lines, relative to the Q7Q7 cells, is not limited to the identified peptides; analysis of all peptides listed in S1 Table (identified and unidentified) showed a similar result.

**Fig 4:**
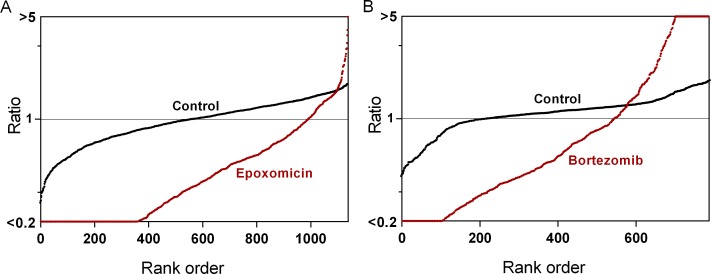
Summary plots of the peptidome of Q7Q7 cells in response to proteasome inhibitors. A: Cells were treated with 200 nM epoxomicin for 1 hour. B: Cells were treated with 500 nM bortezomib for 1 hour. The relative levels of all peptides identified by MS/MS analysis in each of the inhibitor-treated replicates was compared to the average level of the peptide in the untreated control replicates. The *y*-axis represents the relative peptide levels (log-scale) and the *x*-axis represents the rank order of peptides sorted according to the relative level. If the ratio was <0.20 or >5.0, the value was capped at 0.20 or 5.0 to reflect the typical signal to noise ratio. Red circles indicate the ratio of each replicate of identified peptides in inhibitor-treated cells, expressed relative to the average control value. Black circles indicate the ratio of each control replicate expressed relative to the average control value.

Previous studies on other cell lines have shown that the majority of the endogenous peptides are proteasome products, based on the finding that levels are greatly altered by short-term treatments with proteasome inhibitors such as epoxomicin and bortezomib [33–35]. To determine whether the observed peptides in Q7Q7 cells are products of proteasomal degradation of cellular proteins, the cells were treated with epoxomicin or bortezomib for 1 hour. Control replicates were incubated with 0.05% DMSO for the same length of time. Peptide levels were determined by quantitative peptidomics. The majority of peptides in the Q7Q7 cells showed a decrease with epoxomicin treatment and only a few were elevated (Fig 4A). Bortezomib decreased the levels of many peptides but also increased the levels of some peptides (Fig 4B). Changes in the peptidome of Q7Q7 cells in response to proteasome inhibitors is consistent with the proposal that these peptides are produced by proteasome-mediated cleavage of cellular proteins.

**Fig 5:**
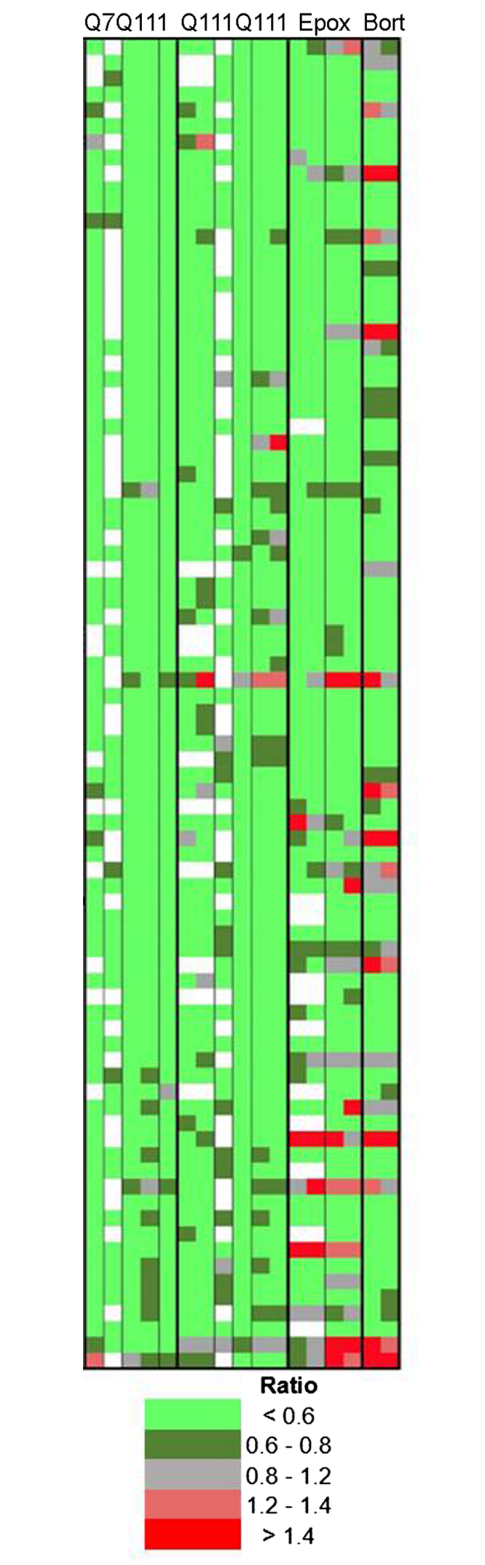
Heat map analysis of selected peptides. Peptides frequently observed in multiple experiments were selected for this analysis. Each row denotes a specific peptide, each column represents a different experiment and each sub-column indicates peptide levels in the biological replicates within that experiment. Ratios of peptides found in multiple charged states and with different tag numbers were averaged together. The ratio was color-coded using the scheme shown at the bottom of the figure, with green representing decreases and red representing increases. Grey represents peptides that did not change substantially. White corresponds to peptides that were either not detected or which could not be accurately quantified due to peak overlap with another co-eluting peptide. Names of proteins, peptide sequences, and peptide ratios are included in S2 Table.

The summary plots in Figs 3 and 4 show all identified peptides listed in S1 Table, with each data point corresponding to a separate replicate. To directly compare peptides in each replicate, we selected a subset of the peptides that were most frequently detected. Of the 341 distinct peptides identified in our study, 32 of these were detected in every experiment, and another 52 detected in nearly every experiment, for a total of 84 commonly detected peptides. Details of these 84 peptides, such as sequence, mass, and cleavage sites, are provided in S2 Table. To visualize the relative levels of each peptide in every replicate, a heat map was created (Fig 5). For these analyses, relative levels of peptides found multiple times with different charge states and/or tag numbers were averaged to provide a single value for each replicate. In the heat map, each row represents a different peptide, and each column an individual experiment, with sub-columns representing the different biological replicates. As with the summary plots shown in Figs 3 and 4, the peptide levels are relative to those found in untreated Q7Q7 cells. Bright green represents peptides that are greatly decreased (ratio <0.60), dark green represents peptides that showed small decreases (ratio 0.60 to 0.80), grey represents peptides that were within 20% of levels in the untreated Q7Q7 cells (ratio >0.80 but <1.20), dark red represents peptides that showed a small increase (ratio 1.20 to 1.40) and bright red represents peptides that showed a large increase (ratio >1.40). White indicates peptides that were not detected in the experiment. In general, the variability of peptide levels in the replicates is low, and most peptides show the same change in each experimental condition. Importantly, the vast majority of peptides that decreased in the Q7Q111 and Q111Q111 cells (relative to Q7Q7 cells) are also decreased by proteasome inhibitor treatment of the Q7Q7 (Fig 5). This suggests that these peptides are products of proteasome cleavage of cellular proteins.

The 32 peptides detected in every experiment were tested for statistical significance (Table 1). All differences >20% between levels in the Q111-expressing cells (heterozygous or homozygous) and the Q7Q7 cells were statistically significant. With only a few exceptions, all differences >20% in response to proteasome inhibitors were also statistically significant (Table 1). None of the peptides that varied less than 20% between untreated Q7Q7 cells and the other groups were statistically significant. Therefore, the quantitative peptidomics approach used in our analysis is generally able to detect differences >20% with confidence, and only a small number of peptides showed variability among the control replicates that prevented statistically significant results.

**Table 1:** Peptides commonly detected in Q7Q7 cells and their relative levels in Q111-expressing cells or proteasome inhibitor treated cells, compared to levels in untreated Q7Q7 cells.

Gene	Peptide	Q7Q111	Q111Q111	Epoxomicin	Bortezomib
Cox5a	GISTPEELGLDKV	0.43±0.18*	0.47±0.10**	≤0.20±0.01***	0.28±0.01**
Eif5a	SAMTEEAAVAIKAMAK	0.37±0.14***	0.44±0.22**	0.36±0.06***	0.35±0.03**
Erh	Ac-SHTILLVQPTKRPEGRTY	0.40±0.07***	0.44±0.14***	0.68±0.03**	0.78±0.06*
Hspe1	AAETVTKGGIMLPEKSQGKVLQA	0.34±0.13***	0.31±0.10***	≤0.20±0.01**	0.31±0.05*
Hspe1	TVVAVGSGGKGKSGEIEPVSV	0.42±0.18**	0.41±0.28*	0.35±0.06**	1.59±0.49*
Lgals1	DLTIKLPDGHEF	0.28±0.10***	0.37±0.19***	≤0.20±0.01***	0.22±0.02***
Lgals1	DLTIKLPDGHEFKF	0.24±0.03***	0.34±0.21***	0.28±0.10***	≤0.20±0.01***
Lgals1	NTKEDGTWGTEHREPAFPFQPGSITEV	0.33±0.19***	0.42±0.19***	0.34±0.17**	0.56±0.11
Lgals1	PDGHEFKFPNRL	0.24±0.06***	0.28±0.10***	0.27±0.05***	0.79±0.08
Lgals1	RGEVASDAKSFVLNL	0.28±0.08***	0.35±0.17***	0.26±0.04***	0.23±0.01**
Lgals1	RGEVASDAKSFVLNLGKDSNNL	0.26±0.01***	0.26±0.07***	0.42±0.19***	0.34±0.06***
Lgals1	SDAKSFVLNL	0.28±0.07***	0.26±0.07***	≤0.20±0.01**	≤0.20±0.01*
Lgals1	SDAKSFVLNLGKDSNNL	0.26±0.04***	0.29±0.11***	0.27±0.05***	0.25±0.04***
Lgals1	TKEDGTWGTEHREPAFPFQPGSITEV	0.35±0.19***	0.47±0.25**	0.38±0.15***	0.50±0.02**
Lgals1	VASDAKSFVLNL	0.25±0.10***	0.25±0.10***	0.55±0.22	0.27±0.02*
Lgals1	VASDAKSFVLNLGKDSNNL	0.24±0.04***	0.27±0.09***	0.95±0.43	0.42±0.14*
Mif	AQATGKPAQYIAVHVVPDQL	0.26±0.06***	0.38±0.18**	0.26±0.08***	0.29±0.05***
Mif	AQATGKPAQYIAVHVVPDQLMTF	0.41±0.16***	0.47±0.21**	0.35±0.07**	0.33±0.04**
Ppia	ADKVPKTAENFRAL	0.34±0.05***	0.53±0.16**	0.23±0.04***	0.63±0.11**
Ppia	EDENFILKHTGPGILSM	0.42±0.09***	0.60±0.21*	0.28±0.10***	0.23±0.01***
Ppia	ELFADKVPKTAENFRAL	0.37±0.05***	0.48±0.14**	0.38±0.10***	0.70±0.01*
Ppia	KTEWLDGKHVVF	0.34±0.09***	0.60±0.23*	0.22±0.03***	0.37±0.04***
Rps21	AKADGIVSKNF	0.41±0.04***	0.40±0.11***	0.24±0.04***	0.31±0.04**
Rps28	Ac-MDTSRVQPIKL	0.44±0.08***	0.44±0.10***	0.21±0.02***	0.35±0.03***
Rps28	VKGPVREGDVLTLLESEREARRLR	0.50±0.09***	0.52±0.19**	0.34±0.17**	0.25±0.06**
Tmsb10	Ac-ADKPDMGEIASFDKAKLKKTETQEKNTLPTKETIEQEKRSEIS	0.51±0.07***	0.87±0.12	1.20±0.42	1.68±0.52**
Vim	LIKTVETRDGQVINETSQ	0.51±0.08***	0.26±0.07***	0.70±0.48	1.07±0.05
Vim	LLIKTVETRDGQVINETSQHHDDLE	0.40±0.10***	0.35±0.14***	1.39±0.14**	0.42±0.08**
Vim	NDRFANYIDKV	0.33±0.08***	0.27±0.08***	0.26±0.07***	0.42±0.01***
Vim	RKLLEGEESRISLPLPTFSSL	0.49±0.12**	0.34±0.09***	0.38±0.22***	0.25±0.01***
Vim	RTLLIKTVETRDGQVINETSQ	0.46±0.17***	0.33±0.18***	0.68±0.51	0.91±0.11
Vim	TLLIKTVETRDGQVINETSQHHDDLE	0.52±0.16***	0.43±0.20***	0.69±0.39	0.35±0.04**

All values are mean ± standard deviation.

*** p≤0.001

** p≤0.01

* p≤0.05 compared to untreated Q7Q7 cells, using Student’s t-test: Q7Q111 (n = 5) and Q111Q111 (n = 6) versus Q7Q7 (n = 4); epoxomicin (n = 4) and bortezomib (n = 2) versus DMSO-treated Q7Q7 cells (n = 6).

The 32 peptides listed in Table 1 are derived from 11 distinct genes. To test if the decrease in peptide levels in the Q111-expressing cells is due to reduced levels of mRNA, we used quantitative PCR to compare levels between the Q7Q7 and Q111Q111 cell lines. Only one of the mRNAs (Lgals1) is significantly lower in Q111Q111 cells, while three (Cox5a, Eif5a, and Rps21) are significantly higher in the Q111Q111 cells, compared to Q7Q7 cells (S2 Fig). The other 7 mRNAs are not significantly different between the cell lines. Therefore, except for peptides derived from Lgals1, the lower levels of peptides reported in Table 1 in the Q111Q111 cells cannot be explained by reduced levels of mRNA. The other 69 genes that gave rise to the hundreds of other peptides detected in this study (which are listed in S1 Table but not in Table 1) were not tested in this analysis.

Another possibility to account for the lower levels of peptides in the Q111-expressing cells, relative to the Q7Q7 cells, would be elevated levels of a downstream peptidase that degrades the peptides in the Q111-expressing cells. Although the enzymes that degrade the intracellular peptides detected in our study have not been conclusively identified, intracellular peptides produced by the proteasome are thought to be degraded by aminopeptidases [43,44]. Because half of the 32 peptides listed in Table 1, and approximately half of the 341 peptides listed in S1 Table have aliphatic residues on the N-terminus, we tested cell extracts with Leu-AMC; this substrate detects both leucine aminopeptidase and puromycin-sensitive aminopeptidase, which cleave a broad range of hydrophobic residues from the N-termini of peptides [45]. Extracts of all three cell lines cleaved the substrate and this cleavage was substantially inhibited by both bestatin and puromycin; this indicates that the major Leu-AMC-cleaving peptidase in all three cell lines is puromycin-sensitive aminopeptidase (which is inhibited by both compounds) and not leucine aminopeptidase (which is inhibited only by bestatin). When normalized to the protein content of the cells, there was no statistically significant difference in the peptidase activity of Q7Q7 and Q111Q111 cells (S3 Fig). The Q7Q111 cells had significantly lower levels of peptidase activity compared to Q7Q7 cells (S3 Fig). Therefore, the lower level of peptides in the Q111-expressing cells is clearly not due to an elevation of puromycin-sensitive aminopeptidase activity in either of the two Q111-expressing cell lines.

**Fig 6:**
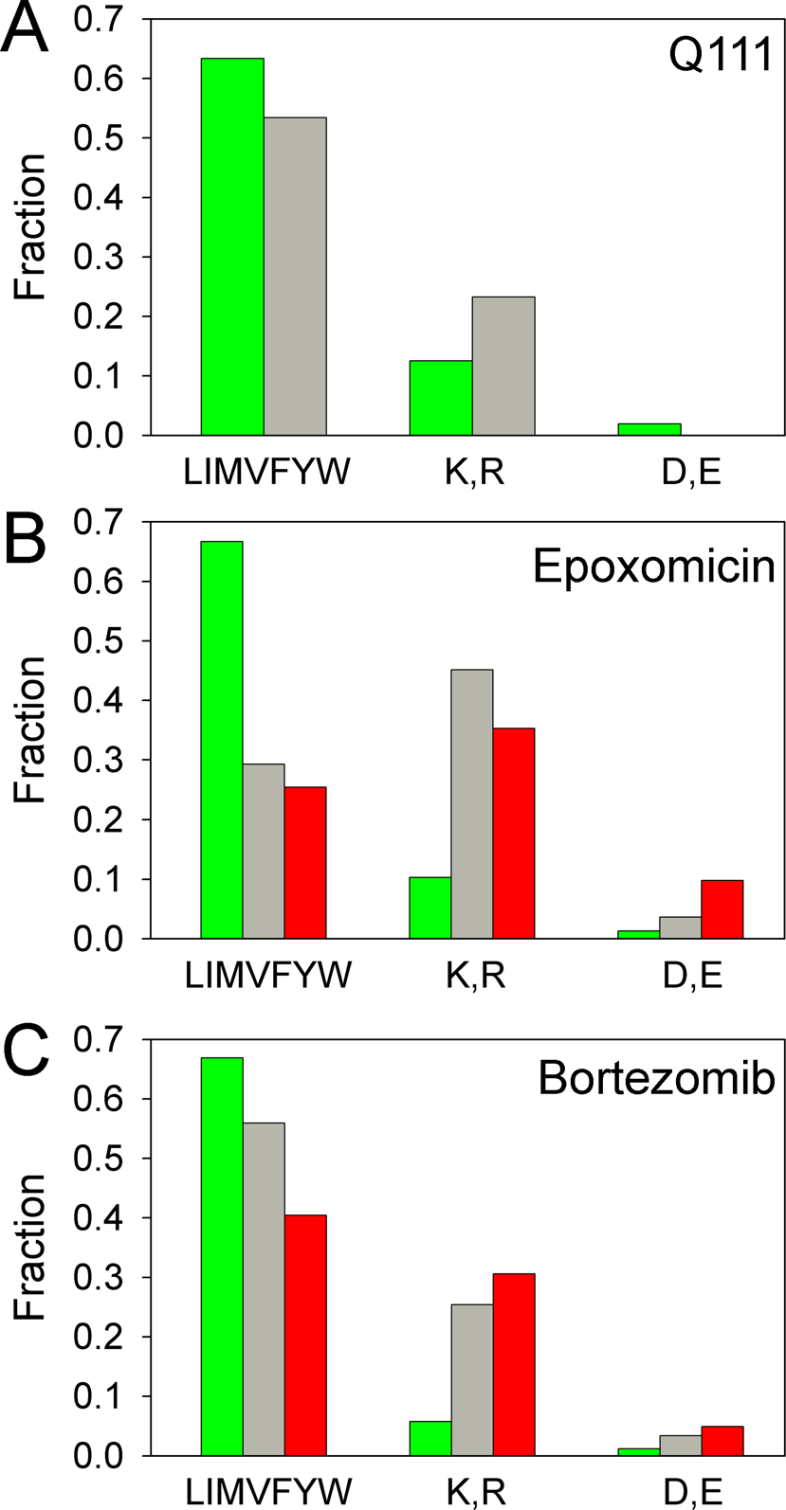
Analysis of amino acids in the P1 position of the cleavage site. All identified peptides in each experiment were grouped into categories based on levels relative to those in untreated Q7Q7 cells. Decrease (green bars) represent ratio ≤0.80, no change (grey bars) represent ratios of 0.81 to 1.24, increase (red bars) represent ratio ≥1.25. The residue in the P1 position of the cleavage site was considered for every peptide; peptides found in multiple replicates were counted each time found. For peptides that represent the N-terminal or C-terminal fragment of the protein, a single cleavage is sufficient to generate the peptide. Peptides that represent internal fragments of proteins require two cleavages, and so the number of cleavage sites is greater than the number of peptides. A: Cleavage site analysis of peptides in Q111-expressing cells. The data for peptides in heterozygous Q7Q111 cells were pooled with the data for peptides in homozygous Q111Q111 cells, resulting in 1441 cleavage sites for peptides that decreased (relative to Q7Q7 cells) and 73 cleavage sites for peptides in the “no change” group. Only 12 cleavage sites from 7 peptides were in the “increased” group, so it was not included in the graph. B: Cleavage site analysis of peptides in Q7Q7 cells treated with 200 nM epoxomicin for 1 hour. Peptides found to decrease represent 775 cleavage sites, the “no change” group represent 82 cleavage sites, and the increase group represents 51 cleavage sites. C: Cleavage site analysis of peptides in Q7Q7 cells treated with 500 nM bortezomib for 1 hour. There were 417 cleavage sites in the group that decreased, 59 cleavage sites in the unchanged group, and 183 cleavage sites in the group that increased after the treatment.

The proteasome inhibitors epoxomicin and bortezomib primarily inhibit the β5 (chymotrypsin-like) proteasome subunit at the concentrations used in our study [46,47]. To determine if the peptides altered by treatment of Q7Q7 cells with proteasome inhibitors were the result of cleavage at sites favored by the β5 subunit, we examined the cleavage sites of all identified peptides listed in S1 Table. We also examined the peptides detected in the studies comparing Q111-expressing cells with Q7Q7 cells, combining heterozygous Q7Q111 and homozygous Q111Q111 data. For this analysis, large and small decreases were grouped together (i.e. ratio ≤0.80). Large and small increases after bortezomib treatment were also grouped together (ratio ≥1.25). Only seven peptides were found to increase in the Q111-expressing cells (Fig 3), so this group was not included in the cleavage site analysis. Peptides found in multiple experiments were counted each time the peptide was observed so that the results would reflect the abundance of the peptides. Those peptides that corresponded to the N- or C-terminus of the protein required only a single cleavage, while those peptides corresponding to internal protein fragments require two cleavages; thus, the number of cleavage sites was greater than the number of peptides. The most commonly observed amino acid in the P1 position of the cleavage site was Leu, representing approximately 30% of all cleavages. Leu, together with other hydrophobic residues Ile, Met, Val, Phe, Tyr, and Trp, was found in the P1 position of approximately 60–70% of all cleavage sites of peptides that decreased either in Q111-expressing cells or after treatment with epoxomicin or bortezomib (Fig 6). For the peptides unaffected by Q111-expression or proteasome inhibitor treatment, less than 55% had hydrophobic cleavage sites. Some peptides elevated by treatment with epoxomicin and bortezomib had hydrophobic cleavage sites (Fig 6). Basic amino acids Lys and Arg were not commonly found in the P1 position of the cleavage site of peptides that were decreased by Q111-expression or treatment with proteasome inhibitors (Fig 6). Acidic amino acids Asp and Glu represent only 2% of all cleavage sites. Peptides that decreased in response to proteasome inhibitors were less likely to have Asp or Glu in the cleavage site, compared to peptides that were unaffected or increased by these treatments (Fig 6B and 6C). In contrast to the effect of proteasome inhibitors, none of the peptides unaffected by Q111-expression had an acidic residue in the cleavage site (Fig 6A).

## Discussion

There are many theories to explain how polyQ tracts in proteins such as huntingtin cause neurodegeneration [2]. One interesting proposal is that long polyQ tracts are not efficiently degraded by the proteasome, leading to general inhibition of protein turnover and eventual cell death [9]. Some evidence supports this theory, such as the finding that proteasomes extracted from Q111Q111 cells have reduced activity towards small synthetic substrates cleaved by the β5 and β1 proteasome subunits [20]. In the present study, we extended this to the heterozygous Q7Q111 cells and found a similar reduction in β5 activity as found in the homozygous Q111Q111 cells. We also confirmed that homozygous Q111Q111 cells have elevated β2 activity using the small synthetic substrates (Fig 1), as was previously reported [20]. Studies using other approaches to examine proteasome activity yielded mixed results, with some showing impairment and others showing normal proteasome activity in the presence of long polyQ tracts [9–28]. Each of these previous studies used a different method, such as introduction of a reporter protein that is rapidly degraded by proteasomes, or active site-directed affinity labels. In all cases, these previous studies required manipulation of the cells, either by extracting proteasomes and measuring activity, or by adding a reagent to the cells to detect proteasome function. Our novel approach uses peptidomics to measure levels of endogenous peptides present in cells. In this approach, cells are heat-inactivated prior to extraction of peptides and subsequent steps. Thus, peptide levels reflect those in normal cells without the addition of reporter proteins or other reagents. The large number of peptides detected in our assay provides a reflection of proteasome activity toward a variety of proteins, not a single reporter construct. The major finding of the present study is that the two cell lines expressing long polyQ tracts have decreased levels of most intracellular peptides, compared to the levels of these peptides in Q7Q7 cells. This supports the hypothesis that proteasome function is impaired by long polyQ tracts.

An important question is why the different approaches to measure proteasome activity in the presence of long polyQ tracts give different results. Most of the other studies used a small number of substrates or active site probes, and it is possible that each of these approaches measures a different form of the proteasome. The proteasome is a protein complex that includes a core 20S particle that is a dimer of 7 α and 7 β subunits, for a total of 28 subunits arranged in a tube-like structure [7,8,48,49]. The three catalytically active β subunits (1, 2, and 5) are replaced by related subunits in specific cells of the immune system, thymocytes, or spermatocytes [50]. In addition, there is heterogeneity in the proteins that associate with the 20S proteasome core; both ends of the tube-like structure of the 20S core can associate with another complex, termed a cap, and there are three different types of cap complexes in mammalian cells [51–53]. To add further complexity, proteasomes exist with no caps, caps on just one end, and caps on both ends—either the same or different. Because the various proteasome forms are optimal towards different substrates, each proteasome assay is likely to detect only a subset of the total proteasome activity [51–53]. Although our broad approach of measuring hundreds of endogenous peptides is theoretically more likely to reflect multiple proteasome activities, compared to assays that use a single substrate, it is not known if these endogenous peptides are primarily generated by the 20S or 26S proteasome, or another one of the forms.

The endogenous peptides present in the striatal cell line are likely to be proteasome products based on the finding that most of these peptides were affected by treatment of the cells with proteasome inhibitors. These results are consistent with previous studies in HEK293T and SH-SY5Y cells with bortezomib, epoxomicin, and other proteasome inhibitors [33–35]. In all three cell lines, some peptides decrease while others are elevated by treatment with proteasome inhibitor. Because the length of treatment with proteasome inhibitors in the previous and present studies were short (30–60 minutes), it is unlikely that the changes in peptide levels were due to altered transcription or translation, which typically takes hours to alter protein levels [47,54–59]. A more likely explanation for the increased levels of some peptides has to do with the fact that bortezomib and epoxomicin primarily inhibit the β5 subunit at the concentrations used in the present study, and not the β1 or β2 activities [47]. In this scenario, proteins that are loaded into the inner chamber of the proteasome remain trapped until cleaved. If the β5 subunit is inhibited by epoxomicin or bortezomib, then the other subunits will play a more major role, and their products will increase. Cleavage site analysis (Fig 6) partially supports this, but there are many peptides predicted to be produced by the β5 subunit that are elevated by bortezomib treatment. Bortezomib is known to inhibit cellular peptidases, and this inhibition could potentially block the degradation of intracellular peptides and result in elevated levels of these peptides [33,60]. However, none of the known off-target effects of bortezomib are likely to occur at the relatively low concentrations that produce elevated levels of peptides; 500 nM in the present study, and 50 nM in a previous study on HEK293T cells [34].

Most of the previous studies on the striatal cell lines used in the present study focused on differences between the Q7Q7 and Q111Q111 cells, and did not analyze the heterozygous Q7Q111 cells [20,61–63]. HD pathology has been shown to result from a toxic gain of function from huntingtin with long polyQ tracts [64]. In HD patients carrying *HTT* alleles of differing CAG repeat number, the longest expanded allele correlates with age at disease onset independent of the other allele’s CAG repeat number [65]. Our finding that Q7Q111 and Q111Q111 cells show comparable levels of most intracellular peptides, which are generally lower than in Q7Q7 cells, supports the toxic gain of function hypothesis [64]. One notable exception is thymosin β-10, which shows a significant decrease in Q7Q111 cells relative to Q7Q7 cells, but is not significantly lower in the Q111Q111 cells (Table 1). Thymosin β-10 is a small protein of 43 amino acids present in many cell types. Because thymosin β-10 does not require proteasome processing, its levels do not reflect proteasome activity and levels are not altered by treatment of Q7Q7 cells with epoxomicin (Table 1). Although levels of thymosin β-10 in Q7Q7 cells were elevated by bortezomib in the present study, this protein was not found to be altered by treatment of HEK293T or SH-SY5Y cells with similar concentrations of bortezomib [33–35].

In addition to the long polyQ tract, the huntingtin protein contains a region rich in proline immediately downstream of the polyQ tract. None of the proteasome subunits are thought to efficiently cleave proteins adjacent to either glutamine or proline, and the combined stretch of these residues in huntingtin protein may be especially inhibitory. In the original proposal, the polyQ stretches were thought to clog the inner chamber of the proteasome, blocking protein entry which would affect all cleavages [9]. However, expression of long polyQ appears to cause a greater decrease in β5 activity than β2 activity, based on a previous study that examined cleavage of synthetic substrates by extracts of Q7Q7 and Q111Q111 cells [20] as well as our analysis of these cells. Furthermore, while the heterozygous Q7Q111 cells have reduced β5 activity, the level of β2 activity is similar to the level in Q7Q7 cells (Fig 1). Taken together with the results from the analysis of cleavage sites that generate peptides detected in the present study (Fig 6), the long polyQ inhibits β5 activity more than β2 activity. It is not clear as to how long polyQ tracts can selectively inhibit β5. One model to explain the apparent activation of β2 activity by bortezomib involves allosteric regulation of proteasome activity [33], and it is possible that the long polyQ tract also contributes to allosteric changes.

Our finding that levels of endogenous peptides are affected by the presence of long polyQ tracts in huntingtin protein raises another potential model to account for the cell death observed in HD. This new model is based on the concept that intracellular peptides are biologically active, affecting protein-protein interactions and/or other cellular functions [30]. Altered levels of intracellular peptides that are functional could in turn cause widespread cellular changes and contribute to neurodegeneration. This potential role for intracellular peptides is similar to the concept of microRNA, in both cases a small oligomer interacts with a larger polymer and affects its functions [30]. Some evidence suggests that endogenous intracellular peptides are biologically active [66–70]. There is no question that small peptides of 10–20 residues can be functional; numerous studies have found that synthetic peptides perturb protein-protein interactions and affect cellular functions, although it is not clear if most of these peptides are endogenous to cells [71–77]. Previous models of the impact of proteasome inhibition considered only the effect on protein turnover; it was assumed that the peptides produced by proteasomal cleavages would be rapidly degraded by cellular peptidases. The peptides detected in the present study on mouse striatal cells are similar to peptides detected in mouse brain and other tissues, and in human cell lines. These peptides represent a small minority of the proteasomal output, implying that they are protected from degradation, possibly because they are bound to proteins. If functional, their altered levels in cells expressing long polyQ tracts could contribute to cellular changes, possibly in combination with other mechanisms.

## Supporting Information

S1 Fig Labeling scheme for the four LC-MS runs comparing peptide levels between the three cell lines. Peptides were extracted from each cell line and labeled with TMAB tags. A different labeling scheme was used for each experiment to normalize for any differences that may arise from chemical properties of the individual TMAB tags. After labeling, the peptides were pooled and processed for mass spectrometry analysis, as described in Materials and Methods.
(TIF)

S2 Fig Transcript levels in Q111Q111 cells as compared to those in Q7Q7 cells for the eleven genes of the commonly detected peptides listed in Table 1. Levels of mRNA were determined by RT-qPCR and normalized to *Actb*. Error bars show standard error of mean (n = 4 biological replicates). **p < 0.005; *p < 0.05; ns, not statistically significant (p > 0.05), as determined by a two-tailed, unpaired t-test. Bottom panel shows sequences of primers used for RT-qPCR.
(TIF)

S3 Fig Measurement of aminopeptidase activity in Q7Q7, Q7Q111, and Q111Q111 cells. A, B: Cell extracts were treated with the indicated concentrations of bestatin (panel A) or puromycin (panel B) for 30 minutes followed by the addition of Leu-AMC and incubation for 1 hour at 37°C. Enzyme activity was determined by fluorescence measurement of AMC and expressed as percent enzyme activity relative to the control without inhibitor. Error bars show standard error of mean (n = 3), points without error bars had error ranges smaller than the symbol size. C: Cell extracts were incubated with Leu-AMC for 1 hour at 37°C and enzyme activity was determined by fluorescence measurement of AMC. Enzyme activity was normalized to the amount of protein in each cell extract to permit comparison among cell lines. Error bars show standard error of mean (n = 5). Statistical analysis was performed using Student’s t-test: ***, p ≤ 0.001; ns, no significant difference (p > 0.05) versus Q7Q7 cells.
(TIF)

S1 Table All peptides detected in each of the LC-MS runs, and relative levels among replicates. (XLSX)

S2 Table Sequences of the peptides selected for heat-map analysis (Fig 5) and their relative levels in each experiment. (XLSX)
