# Supplementary figures and images for: Reduced Levels of Proteasome Products in a Mouse Striatal Cell Model of Huntington’s Disease
Source: PLoS One. 2015 Dec 21;10(12):e0145333. doi: 10.1371/journal.pone.0145333 (PMC4686214; doi:10.1371/journal.pone.0145333)

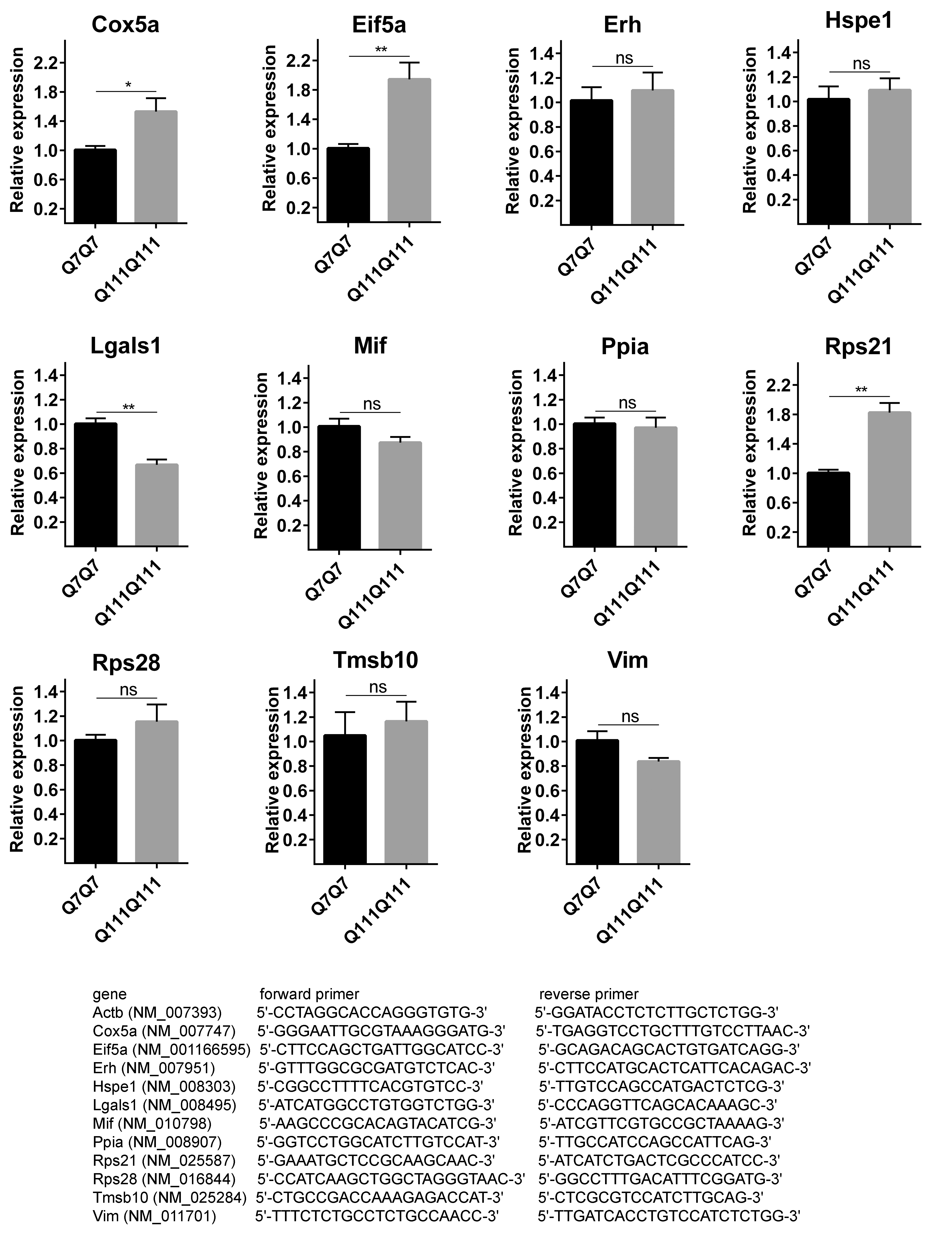

Supplement: S2 Fig — Levels of mRNA were determined by RT-qPCR and normalized to Actb. Error bars show standard error of mean (n = 4 biological replicates). **p < 0.005; *p < 0.05; ns, not statistically significant (p > 0.05), as determined by a two-tailed, unpaired t-test. Bottom panel shows sequences of primers used for RT-qPCR. (TIF) [file pone.0145333.s002.tif]

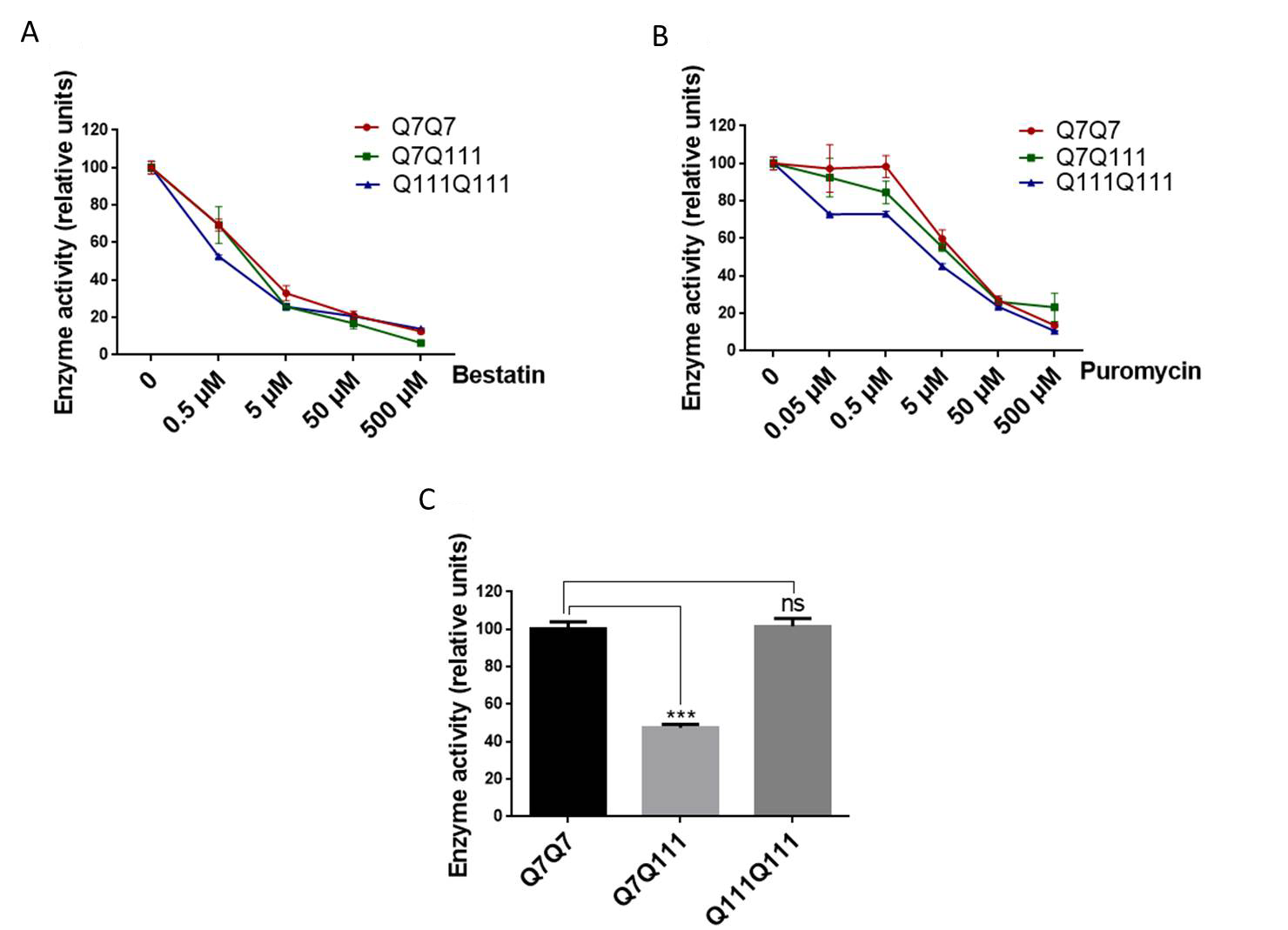

Supplement: S3 Fig — A, B: Cell extracts were treated with the indicated concentrations of bestatin (panel A) or puromycin (panel B) for 30 minutes followed by the addition of Leu-AMC and incubation for 1 hour at 37°C. Enzyme activity was determined by fluorescence measurement of AMC and expressed as percent enzyme activity relative to the control without inhibitor. Error bars show standard error of mean (n = 3), points without error bars had error ranges smaller than the symbol size. C: Cell extracts were incubated with Leu-AMC for 1 hour at 37°C and enzyme activity was determined by fluorescence measurement of AMC. Enzyme activity was normalized to the amount of protein in each cell extract to permit comparison among cell lines. Error bars show standard error of mean (n = 5). Statistical analysis was performed using Student’s t-test: ***, p ≤ 0.001; ns, no significant difference (p > 0.05) versus Q7Q7 cells. (TIF) [file pone.0145333.s003.tif]
